# MRI tumor imaging by click chemistry using Gd(iii)-DOTA-DBCO and Ac_4_ManNAz

**DOI:** 10.1039/d6ra02364a

**Published:** 2026-05-18

**Authors:** Nobuhiko Ichiki, Hisao Saneyoshi, Hiroyuki Satoh, Atsushi Nanashima, Yan Xu

**Affiliations:** a Graduate School of Medicine and Veterinary Medicine, University of Miyazaki 5200 Kihara, Kiyo-take Miyazaki 889-1692 Japan xuyan@med.miyazaki-u.ac.jp; b Division of Chemistry, Department of Medical Sciences, Faculty of Medicine, University of Miyazaki 5200 Kihara, Kiyotake Miyazaki 889-1692 Japan; c Faculty of Agriculture Region of Veterinary Science, University of Miyazaki 1-1 Gakuen Konohanadai Nishi Miyazaki 889-1692 Japan

## Abstract

Magnetic resonance imaging (MRI) is an important diagnostic tool widely used in clinical practice. It does not involve harmful ionizing radiation, allowing for more frequent or long-term imaging. However, MRI suffers from limited sensitivity, and contrast agents are often required to enhance signal intensity and image contrast. In particular, tumors cannot always be clearly distinguished from surrounding tissues. Therefore, one potential strategy to improve MRI for cancer diagnosis is to enable tumor-specific detection. In this study, we synthesized a novel MRI contrast agent by conjugating a gadolinium(iii)-DOTA complex with dibenzocyclooctyne (DBCO). An azide-modified mannosamine derivative was employed for metabolic glycoengineering, enabling the expression of chemical tags on cancer cell surfaces. These tags undergo a bioorthogonal click reaction with the functionalized gadolinium(iii)-DOTA complex, facilitating rapid and sensitive enhancement of MRI contrast for imaging of cancer cells both *in vitro* and *in vivo*.

## Abstract 2

MRI tumor imaging by click chemistry.

## Graphical abstract

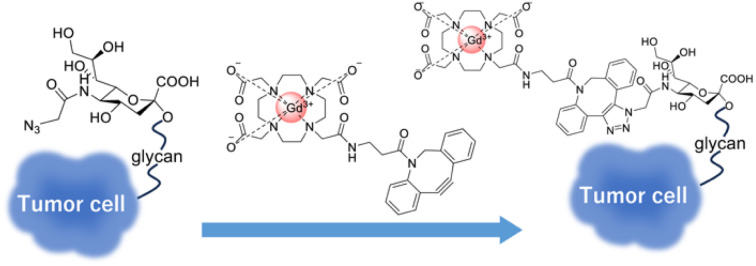

Various imaging techniques, such as magnetic resonance imaging (MRI), computed tomography (CT), and positron emission tomography (PET), have been clinically applied in recent years. Among these, MRI has become widely used due to its advantages, including the absence of radiation exposure, deep penetration into soft tissues, high spatiotemporal resolution, and ease of operation.^1,2^ Currently, commonly used MRI contrast agents enhance diagnostic performance by modifying the water proton relaxation rate of target tissues, thereby increasing the image contrast of surrounding tissues.^3,4^ However, they are not well suited for the early detection of small pathological changes, such as early-stage cancer, and no clinically approved contrast agent provides truly tumor-specific contrast.^5–8^

In this work, we utilized copper-free “click” chemistry to overcome existing limitations and achieve specific tumor imaging through MRI signal amplification both *in vitro* and *in vivo*.^9–14^ Metabolic glycan labeling with unnatural sugars has emerged as a simple and powerful method for tagging cell membranes with chemical handles.^15–23^ Because tumor cells are metabolically active, they exhibit higher uptake of glucosamine derivatives than normal cells, thereby reflecting cell proliferation.^15^ This technology has been widely explored for cancer labeling and targeting; for example, we have previously applied it for fluorescence imaging *in vitro* and *in vivo*.^20^

Here, we first synthesized a clickable gadolinium(iii)-DOTA complex, Gd-DOTA-DBCO, which reacts with azide groups under copper-free conditions (Fig. S1 and 1). A modified mannosamine derivative (Ac_4_ManNAz) can be fed to cells and incorporated into membrane sialoglycoconjugates. Once incorporated, click reactions with Gd-DOTA-DBCO enable labeling of the cell surface. To confirm the click reaction between Gd-DOTA-DBCO and Ac_4_ManNAz, the two compounds were mixed at room temperature for 1 h (Fig. S2) Completion of the reaction within 1 h suggests that bioorthogonal click chemistry may enable efficient binding of Gd-DOTA complexes to cancer cells.

**Fig. 1:**
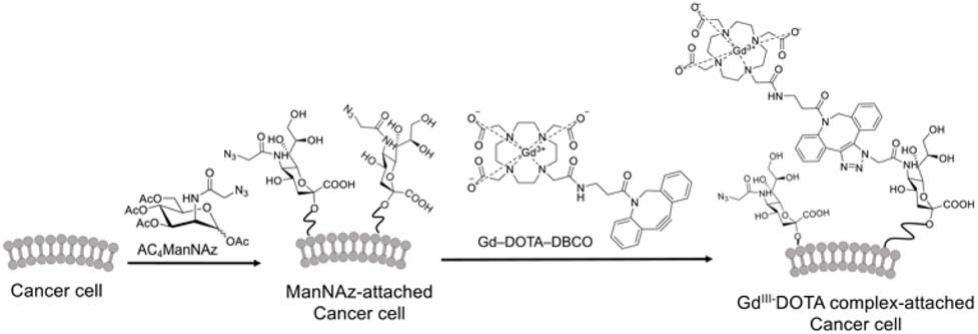
Schematic illustration of the attachment of a Gd(iii)-DOTA contrast agent to the tumor cell surface *via* click chemistry. The Ac_4_ManNAz analogue is supplied to cells and incorporated into membrane sialoglycoconjugates. Following successful incorporation of the modified sugar into the glycoconjugates, a click reaction with Gd-DOTA-DBCO is performed to label the cells.

After confirming the efficient click reaction between the Gd complex and Ac_4_ManNAz, we performed inductively coupled plasma mass spectrometry (ICP-MS) to quantify cellular Gd content (Fig. 2). To evaluate uptake, 4T1 cells were cultured with or without Ac_4_ManNAz and then treated with Gd-DOTA-DBCO. ICP-MS revealed an approximately eightfold increase in Gd accumulation in Ac_4_ManNAz-treated cells compared with untreated controls. These results indicate that Ac_4_ManNAz-treated cells form glycan chains on the plasma membrane that react with the Gd complex *via* click chemistry, firmly anchoring gadolinium on the cell surface and thereby enabling the detection of high Gd levels.

**Fig. 2:**
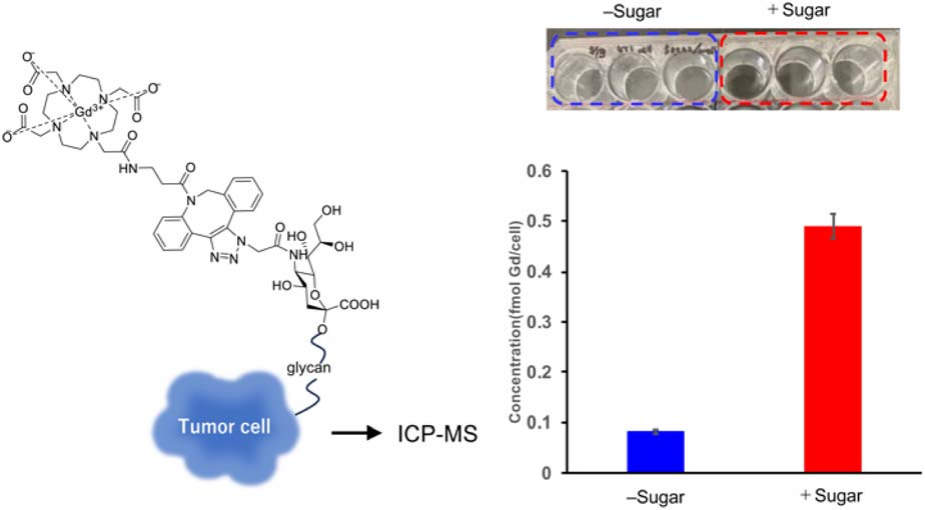
Gd content measured by ICP-MS. 4T1 cells were treated with Ac_4_ManNAz or with PBS alone (without Ac_4_ManNAz) for 3 days. Cells cultured with or without the sugar analogue were then reacted with the Gd(iii)-DOTA complex for 1 h. After washing with PBS, the cells were analyzed by ICP-MS. Quantification of Gd concentration is shown. Data are presented as mean ± s.d. (*n* = 3). The photograph shows the cells incubated in a culture plate.

To evaluate *in vivo* efficacy, a 4T1 tumor-bearing nude mouse model was used (Fig. 3). Ac_4_ManNAz was injected intratumorally for three consecutive days, with PBS used as a control. On day 4, mice were treated with Gd–DOTA–DBCO, after which the tumors were excised and analyzed by MRI. As shown in Fig. 3B, the MR signal enhancement in Ac_4_ManNAz-treated tumors was significantly higher than that in the control group. Histological images confirmed the presence and morphology of tumor tissue.

**Fig. 3:**
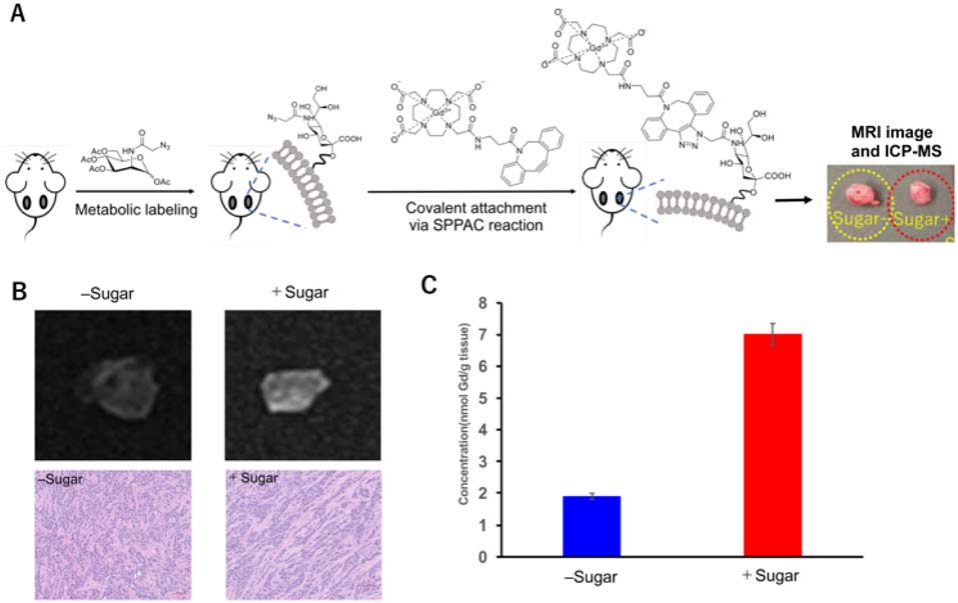
(A) Schematic representation of the attachment of the Gd(iii)-DOTA complex to the tumor surface *in vivo*. Mice bearing 4T1 tumors were treated intratumorally with Ac_4_ManNAz or PBS, followed by administration of Gd-DOTA-DBCO. Tumors were then analyzed by MRI and ICP-MS. The photograph shows tumors with and without Ac_4_ManNAz treatment. (B) (Upper) T1-weighted MR images of tumor tissues treated with or without the sugar analogue after reaction with the Gd(iii)-DOTA complex. (Lower) Histological microscopic analysis of tumor tissues with the corresponding H&E-stained sections. (C) Gd content in tumor tissues measured by ICP-MS. Quantification of Gd concentration is shown. Data are presented as mean ± s.d. (*n* = 3).

Following MRI acquisition, the intratumoral concentration of Gd was then quantified by ICP-MS (Fig. 3C). Ac_4_ManNAz-pretreated tumors exhibited approximately fivefold higher Gd accumulation compared with controls. This enhancement is attributed to Ac_4_ManNAz-derived glycans on tumor cell membranes that undergo copper-free click reaction with the Gd complex, thereby anchoring Gd on the cell surface and resulting in greater retention.

The longitudinal relaxation rate (1/*T*_1_) of water protons were measured to assess relaxivity. The longitudinal relaxivity (*r*_1_) of Gd-DOTA-DBCO was determined to be 3.28 mM^−1^ s^−1^, while that of the clicked complex was 3.72 mM^−1^ s^−1^ (Fig. S3). Although a slightly higher value was observed after the click reaction, the overall relaxivity remained comparable.

Based on these findings, MRI examinations were performed in live mice to assess the temporal dynamics of contrast enhancement. The same glycoengineering treatment described above was applied, followed by intratumoral administration of the Gd-DOTA-DBCO. MRI scans were subsequently acquired at 30 minutes, 1 hour, 2 hours, and 24 hours post-injection. Fig. 4 presents T1-weighted three-dimensional maximum intensity projection images as well as two-dimensional axial images obtained before and after administration of the Gd-complex. In comparison with the PBS-treated controls, the AC_4_ManNAz-treated group exhibited sustained and pronounced enhancement in tumor tissue for at least 2 hours. Notably, retention of the contrast agent was still observed within the tumor at 24 hours, indicating specific association of the agent with tumor tissue. By contrast, in the PBS-treated group, the enhancement gradually diminished over time, and no appreciable enhancement was detectable at 24 hours. The analysis of signal intensity is consistent with the results (Fig. S4). Collectively, these data confirm that *in vivo*, glycoengineering facilitated a bioorthogonal click reaction between the Gd-based contrast agent and the unnatural sugar, thereby enabling prolonged retention of Gd within tumor tissue.

**Fig. 4:**
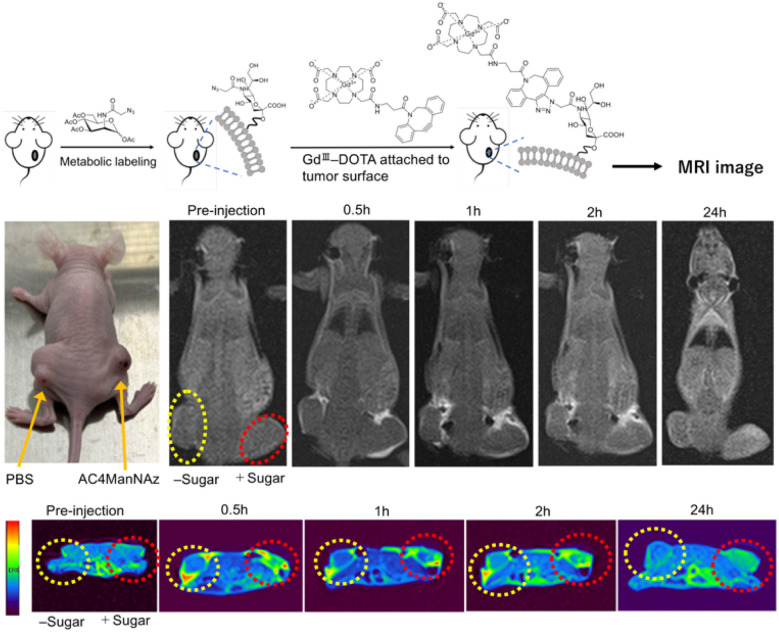
Schematic illustration and representative T1-weighted MR images showing the experimental workflow and imaging results. Tumor-bearing mice were pretreated with the azido sugar analogue AC_4_ManNAz (+Sugar) or PBS (−Sugar), followed by intravenous administration of Gd-DOTA-DBCO. Bioorthogonal click chemistry enables selective attachment of the Gd-DOTA complex to azide-labeled glycans on the tumor cell surface. T1-weighted MR images were acquired before injection and at 0.5, 1, 2, and 24 h post-injection. Enhanced and prolonged T1 signal intensity was observed in tumors pretreated with Ac_4_ManNAz compared with the PBS control, indicating successful tumor-surface labeling and retention of the Gd-DOTA contrast agent. The upper images are whole-body T1-weighted MR images, shown in grayscale, to allow comparison of global signal distribution. The lower images are magnified tumor-region images with pseudo-color overlay, used to emphasize local T1 signal enhancement at the tumor site.

At 24 h post-administration, gadolinium was scarcely detected in the heart, lungs, and spleen (Fig. S5). In contrast, relatively high accumulation was observed in the liver and kidneys. Notably, a comparatively high level of gadolinium was also detected in tumor tissue. These results suggest that, although the contrast agent was locally administered into the tumor, a portion of the gadolinium complex entered systemic circulation and was subsequently distributed to major clearance organs. The high accumulation in the kidneys is consistent with renal excretion pathways (*e.g.*, glomerular filtration and tubular processes), while liver uptake may reflect involvement of the reticuloendothelial system and metabolic processing. The retention of gadolinium in tumor tissue at 24 h may be attributed to its immobilization on the cell surface *via* metabolic glycoengineering.

In the present study, we synthesized a novel MRI contrast agent based on a bioorthogonal click chemistry approach combined with metabolic labeling. Metabolic glycoengineering (MGE) with unnatural monosaccharides has emerged as a facile yet powerful strategy for cellular labeling and visualization. Positron emission tomography (PET) using the 18F-FDG radionuclide glucose is a representative diagnostic imaging modality derived from MGE. However, to date, no reports have demonstrated the application of MGE in the development of MRI contrast agents.

The cell-surface presentation of the azide-functionalized mannosamine derivative in this study is inferred based on well-established metabolic glycoengineering mechanisms reported in previous studies. These studies have shown that modified monosaccharides are taken up by cells, metabolically incorporated, and expressed on the cell surface as glycoconjugates. Thus, we expect a similar process to occur in our system.

When used in conjunction with azido-sugar derivatives, this agent enables rapid and sensitive cellular imaging both *in vitro* and *in vivo*, as well as tumor-specific imaging in tumor-bearing mice. Our findings suggest that tumor-specific MRI imaging may provide a basis for tumor detection. This encourages continued research toward the potential development of MRI contrast agents capable of achieving tumor-specific imaging in clinical practice.

Although intratumoral injection is not clinically standard, it was used here as a proof-of-concept to directly evaluate the feasibility of the *in situ* bioorthogonal click reaction under controlled conditions. This approach reduces systemic effects and allows clearer assessment of the targeting mechanism. The results demonstrate effective local MRI signal enhancement at the tumor site. However, this method limits clinical relevance. Future studies will focus on systemic administration (*e.g.*, intravenous injection) to evaluate tumor targeting under more realistic conditions. These experiments require further optimization and are left for future work.

## Ethical statement

All animal experiments were conducted in accordance with relevant laws and regulations and were approved by the Institutional Animal Care and Use Committee of the University of Miyazaki (Miyazaki, Japan).

## Conflicts of interest

There are no conflicts to declare.

## Supplementary Material

RA-016-D6RA02364A-s001

## Data Availability

The data supporting this article have been included as part of the supplementary information (SI). Supplementary information is available. See DOI: https://doi.org/10.1039/d6ra02364a.
