# Supplementary material for: MRI tumor imaging by click chemistry using Gd(iii)-DOTA-DBCO and Ac4ManNAz
Source: RSC Adv. 2026 May 18;16(29):26448–51. doi: 10.1039/d6ra02364a (PMC13185799; doi:10.1039/d6ra02364a)
Supplement: RA-016-D6RA02364A-s001 [file RA-016-D6RA02364A-s001.pdf]

Supplementary Information (SI) for RSC Advances.
This journal is © The Royal Society of Chemistry 2026

Supporting Information

Preparation of Gd–DOTA–DBCO complex

DO3A–DBCO (23 mg, 29.6 μmol) was dissolved in H_2O (5 mL) at room temperature. An excess of $Gd(OH)_3$ hydrate (62 mg, 235 μmol) was added, and the pH of the solution was adjusted to 6 using 1.0 M NaOH. The reaction mixture was stirred for 5 min while monitoring the pH. The resulting solution was purified by HPLC (Solvent A: H_2O containing 0.1% TFA; Solvent B: acetonitrile; gradient: 40% B for 20 min, then 50% B for 20 min). After solvent removal, the Gd complex was obtained as a clear oil and subsequently lyophilized to afford a fluffy white solid (22 mg, 27.0 μmol, 92% yield). HRESI-MS for $C_{34}H_{40}GdN_6O_8^+$: Calcd. 818.2143, Found 818.2234.

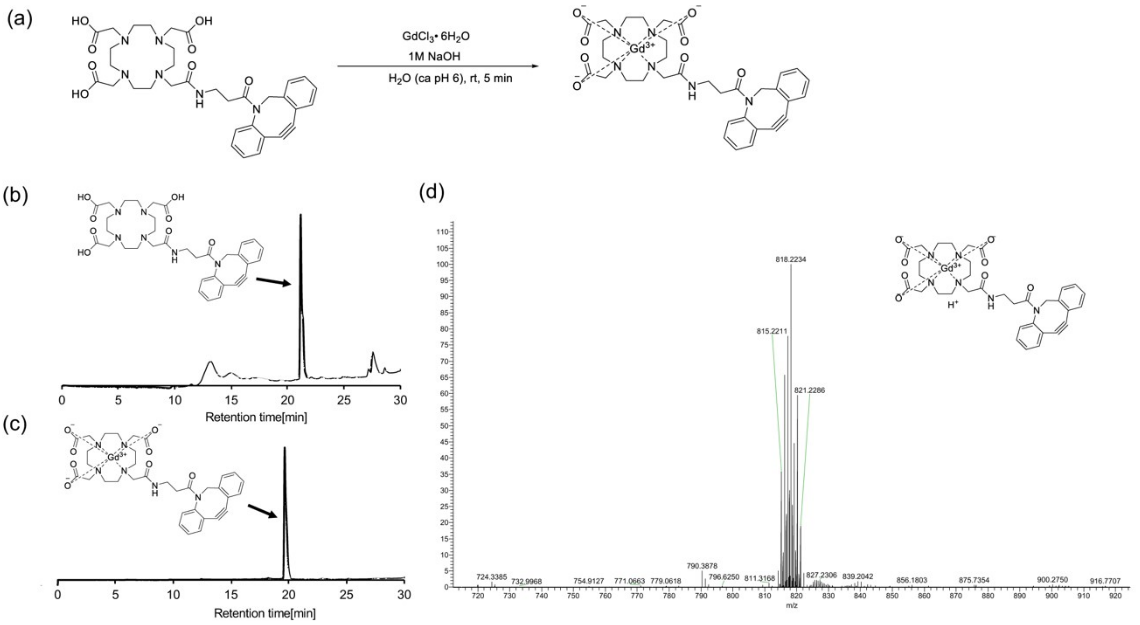

Figure S1. (a) Synthetic route Gd–DOTA–DBCO. (b, c, d) The reaction product was purified by HPLC and confirmed by high-resolution electrospray ionization mass spectrometry (HRESI-MS).

Click reaction between $Ac_4ManNAz$ and Gd–DOTA–DBCO

The Gd complex (0.0816 mg, 100 nmol) was mixed with AC4ManNAz (10 mM in DMSO, 50 µL, 500 nmol) at room temperature. After 1 h, the reaction mixture was purified by HPLC and analyzed by HRESI-MS. HRESI-MS for $C_{50}H_{62}GdN_{10}O_{18}^{+}$: Calcd. 1248.3479, Found 1248.3628.

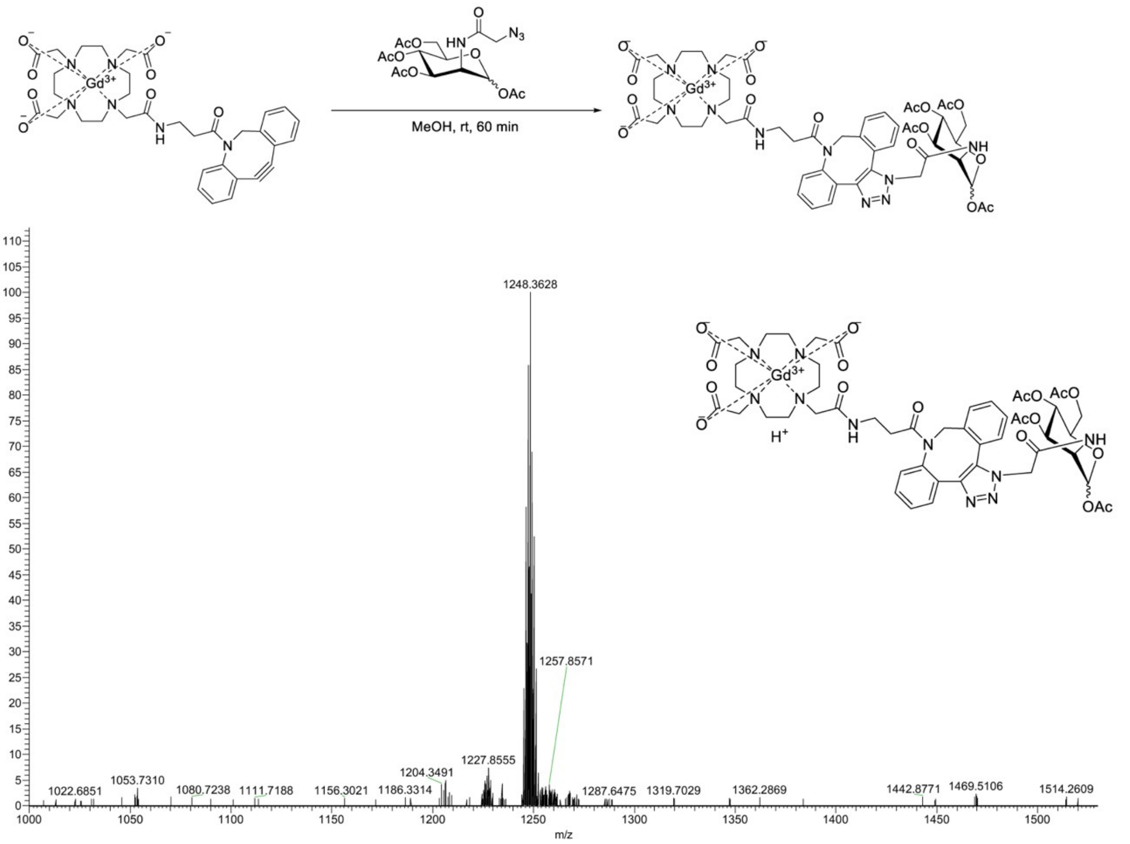

Figure S2. Click reaction between Gd–DOTA–DBCO and $Ac_4ManNAz$._The reaction product was purified by HPLC and confirmed by high-resolution electrospray ionization mass spectrometry (HRESI-MS)

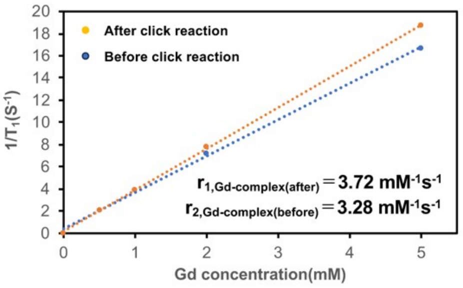

Figure S3. Water proton longitudinal relaxation rate (1/T1) of Gd-complex (before and after click reaction) in aqueous solution as a function of Gd concentration

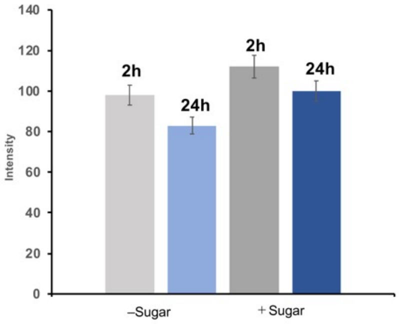

Figure S4. The corresponding MRI signal intensities at 2 and 24 h in Ac_4ManNAz-treated and control tumors. Data are presented as mean ± s.d. (n = 3).

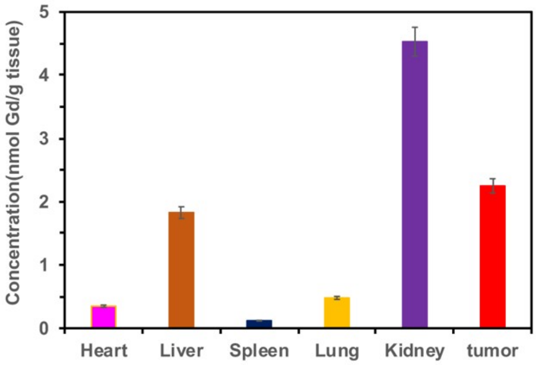

Figure S5. Biodistribution of Gd in various tissue after 24 h into nude mice. Data are presented as mean ± s.d. (n = 3).

Cell experiments

Approximately 5 × 10^4 cells were seeded into each well of a 24-well flat-bottom plate and incubated for 3 days Ac_4ManNAz (50 μM, 1 mL, mixed solvent of DMSO:DMEM = 5:995, v/v) was administered once daily for three consecutive days to one side. PBS was administered to the other side. On the fourth day, a mixed solvent solution of a gadolinium complex (10 μM, 1 mL, mixed solvent of DMSO:DMEM = 1:999, v/v) was administered. One hour later, the area was washed three times with PBS. RIPA buffer (150 μL) was added to each well, and the plate was incubated on ice for 30 min. Prior to ICP-MS analysis, the cell lysates were digested with an equal volume of concentrated nitric acid at 90 °C for 1 h. Calibration curves were prepared before each analysis using standard solutions with known metal ion concentrations (10, 5, 1, 0.5, and 0 nM). Elemental analysis of Gd was performed using inductively coupled plasma mass spectrometry (ICP-MS).

In vivo MRI imaging of tumor-bearing mice

All animal experiments were conducted in accordance with relevant laws and regulations and were approved by the Institutional Animal Care and Use Committee of the University of Miyazaki (Miyazaki, Japan). BALB/c nude mice (6 weeks old) were purchased and, six days after arrival, 4T1 cells (3.2 × 10^6 cells) were subcutaneously injected into both thighs. Ac_4ManNAz (25 mM, 50 μL, mixed solvent of DMSO:H_2O = 1:9, v/v) was intratumorally injected into the right-side tumor once daily for three consecutive days (days 1–3). The left-side tumor received intratumoral injections of PBS as a control. On day 4, a mixed solvent solution of the Gd–DOTA–DBCO (5 mg/kg : 0.1mg, DMSO:H_2O = 1:9, v/v) was intratumorally injected. On the following day, mice were sacrificed, and tumors and organs were excised. Tumors were treated with collagenase (50–200 U/mL) and 3 mM $CaCl_2$ in Hank's Balanced Salt Solution at 37 °C. RIPA buffer (150 μL) was added to the tumor suspension and incubated on ice for 30 min. The lysates were digested with an equal volume of concentrated nitric acid at 90 °C for 1 h and analyzed by ICP-MS three days later. Calibration curves were prepared using standard solutions with known metal ion concentrations (100, 50, 20, 10, 5, 1, 0.5, and 0 nM). For *ex vivo* MRI, tumors were excised 2 h after Gd administration and imaged by MRI. For *in vivo* MRI, mice were imaged under inhalation anesthesia at 1 h after Gd administration (pre-injection, 30 min, 1 h, 2 h, and 24 h post-injection). Tumors were subsequently excised, processed as described above, and tumor suspensions were analyzed by ICP-MS. MRI measurements were performed using a 9.4 T BioSpec system equipped with a ^{1}H quadrature volume resonator and a 10 mm surface coil.

Nuclear magnetic resonance (NMR) measurements were performed using a 500 MHz NMR spectrometer (Bruker). Samples were dissolved in a heavy solvent (10% D_2O, 10 mM phosphate

buffer) and loaded into 5-mm NMR tubes. Measurements were conducted at 298 K. ^{1}H NMR spectra were acquired using a standard pulse sequence (zg) with 8 scans. Chemical shifts are expressed in ppm relative to the solvent peak (8 scans). Gd-DOTA-DBCO and Gd-complex were prepared at concentrations of 0.5 mM, 1 mM, 2 mM, and 5 mM and analyzed.

Histological Analysis

Tumor tissues were embedded in optimal cutting temperature (OCT) compound, frozen, and sectioned at a thickness of 5 μm. Sections were fixed with 4% formaldehyde in phosphate-buffered saline and stained with hematoxylin and eosin. For fluorescence analysis, sections were counterstained with DAPI. Micrographs were acquired using an Olympus BX53 light microscope, and image processing for HE staining was performed using Olympus cellSens imaging software.
